# Racial and Sex Differences in the Response to First-Line Antihypertensive Therapy

**DOI:** 10.3389/fcvm.2020.608037

**Published:** 2020-12-17

**Authors:** John S. Clemmer, W. Andrew Pruett, Seth T. Lirette

**Affiliations:** ^1^Department of Physiology and Biophysics, Center for Computational Medicine, University of Mississippi Medical Center, Jackson, MS, United States; ^2^Department of Data Science, John D. Bower School of Population Health, University of Mississippi Medical Center, Jackson, MS, United States

**Keywords:** hypertension, African American, black, race, antihypertensive therapy, first line treatment

## Abstract

**Objective:** As compared to whites, the black population develops hypertension (HTN) at an earlier age, has a greater frequency and severity of HTN, and has poorer control of blood pressure (BP). Traditional practices and treatment efforts have had minor impact on these disparities, with over a 2-fold higher death rate currently for blacks as compared to whites. The University of Mississippi Medical Center (UMC) is located in the southeastern US and the Stroke Belt, which has higher rates of HTN and related diseases as compared to the rest of the country.

**Methods:** We retrospectively analyzed the UMC's Research Data Warehouse, containing >30 million electronic health records from >900,000 patients to determine the initial BP response following the first prescribed antihypertensive drug.

**Results:** There were 5,973 white (45% overall HTN prevalence) and 10,731 black (57% overall HTN prevalence) patients who met criteria for the study. After controlling for age, BMI, and drug dosage, black males were overall less likely to have controlled BP (defined as < 140/90 mmHg) and were associated with smaller falls in BP as compared to whites and black females. Blockers of the renin-angiotensin system (RAS) failed to significantly improve odds of HTN control vs. the untreated group in black patients. However, our data suggests that these drugs do provide significant benefit in blacks when combined with THZ, as compared to untreated and as compared to THZ alone.

**Conclusion:** These data support the use of a single-pill formulation with ARB or ACE inhibitor with a thiazide in blacks for initial first-line HTN therapy and suggests that HTN treatment strategies should consider both race and gender. Our study gives a unique insight into initial antihypertensive responses in actual clinical practice and could have an impact in BP control efficiency in a state with prevalent socioeconomic and racial disparities.

## Introduction

Clinical studies investigating antihypertensive efficacy characterize the black population as a significant predictor of a poor blood pressure (BP) response (1, 2), and there has not been a consistent improvement in the last 20 years (3). Black Americans of all ages have a greater frequency and severity of uncontrolled hypertension (HTN) and greater risks from high BP as compared to the white population (4–8). Black males, in particular, have one of the highest prevalence of HTN (44% nationally) (9), lowest BP control rates (30%) (10), and highest rates of HTN-related deaths (11). The mechanisms responsible for these vulnerabilities and the mechanisms that underlie this racial disparity are unclear.

First-line therapies that include 2 antihypertensives reduce mortality, but a large portion of patients fail to move to combination HTN treatment despite poor initial responses to single treatments (12). Further, there are significant evidence gaps for the impact of race and gender on first-line antihypertensive treatment responses in socioeconomically-burdened populations. This is especially true for those who may be unable to have routine medical appointments for drug titration or therapy changes. The University of Mississippi Medical Center is located in the southeastern US and the Stroke Belt, which has very high rates of obesity, diabetes, and HTN and their related complications as compared to the rest of the country (13–15). Mississippi, in particular, is one of the poorest states and consistently has the highest prevalence of HTN (16) and highest HTN-related deaths in the country (17). One of the major aims of the current analysis was to evaluate cardiovascular responses to antihypertensive monotherapies to give clinical insight into which therapies have the best initial benefits in this population. Additionally, with recent advancements in health care digitalization, a subsequent aim was to demonstrate the use electronic health records in the evaluation of actual clinical practice. We retrospectively analyzed the Research Data Warehouse (RDW) from the University of Mississippi Medical Center (UMC), which contains >30 million electronic health records from >900,000 patients.

## Materials and Methods

### Participants

The RDW is a large de-identified databank continually compiled from discrete fields from UMC's Epic system and is exempt from IRB approval (18). RDW data from 2013 to 2020 were obtained from white and black males and females that were ≥18 years old with a clinical HTN diagnosis. Patients were excluded if there were clinical diagnoses of heart failure or record of digoxin therapy, kidney disease, acute or chronic renal failure, liver failure, or cardiomyopathy. The first recorded administration of a single antihypertensive drug and the follow-up appointment were retrieved. Patients were included if the baseline systolic BP (SBP) or diastolic BP (DBP) were ≥140 mmHg or 90 mmHg, respectively, and there was no previous record of antihypertensive therapy. Patients were excluded if the follow-up appointment was <7 days and >12 months. Patients were classified as taking untreated if they had a clinical HTN diagnosis and ≥140/90 mmHg BP but were not taking any antihypertensive therapy, either at baseline or previous. Controlled HTN was defined as <140/90 mmHg.

Drug therapies that were included in the study were a calcium channel blocker (CCB), thiazide (THZ) diuretic, selective beta blocker (BBs), non-selective beta blocker (BB), angiotensin converting enzyme inhibitor (ACEi), angiotensin receptor blocker (ARB), an ARB and THZ diuretic formulation (ARB + THZ), and an ACEi and THZ diuretic formulation (ACEi + THZ). Potassium-sparing diuretics, loop diuretics, and α-antagonists contained patient numbers <10 in some groups and were not included in the study. The patient group sizes of each drug class and dosages are reported in **Table 2** and stratified by low, medium, and high dosing. Specific drug names, doses, and corresponding patient group sizes are reported in the Supplementary Material.

### Statistical Analysis

Summary statistics were compiled where appropriate. Means, standard deviations, and *t*-tests were used for continuous variables and count percentages, and chi-squared-tests for categorical. 95% CI are presented unless otherwise noted. Exploratory fractional polynomial models were constructed for all outcomes. These revealed that an appropriate modeling framework would consist of making a logarithmic transform on the time an individual was on the drug of interest. A base-2 log transform was used to facilitate interpretability. The time variable was then interacted with drug, race, and sex, resulting in a four-way interaction model that was adjusted for age, BMI, drug dose, and baseline BP. The change in BP was modeled using random slope-intercept mixed models in the Gaussian family. HTN control was modeled with logistic regression. Appropriate linear transformation were performed in order to examine the expected change for each drug class, for each race and sex, at 6 months. All statistical analyses were completed in Stata v.16.1 (StataCorp, College Station, TX).

## Results

### Baseline Characteristics and HTN Prevalence

Of the 115,600 total white patients in the RDW that were ≥18 years old with an office visit and/or recorded BP, 51,939 patients had HTN (45% overall prevalence). Of the 121,884 black patients that met criteria, 69,474 were diagnosed with HTN (57% overall prevalence, *p* < 0.0001 vs. white) (Supplementary Figure A1). Black patients were overall more likely to be female, younger, and have higher BP as compared to whites (Table 1). Additionally, black females were associated with higher BMI as compared to all other groups (Table 1). The group sizes of each drug class separated by dose is reported in Table 2. Black patients were most likely to be prescribed CCB (Table 2) and less likely to be prescribed BB, BBs, ARB, and ACEi as compared to white patients (Supplementary Figure A2).

**Table 1:** Demographics and baseline characteristics in treated white and black patients before therapy (mean ± STD shown).

	**Overall**	**White**	**Black**	**White male**	**White female**	**Black male**	**Black female**
*N*	7572	2409	5163	1068	1341	1677	3486
Age (year)	51 ± 15	55 ± 15	49 ± 14^*^	52 ± 14	58 ± 15^+^	49 ± 13	49 ± 14
SBP (mmHg)	154 ± 15	153 ± 15	154 ± 16^*^	152 ± 14	154 ± 15^+^	154 ± 15	154 ± 16^+^
DBP (mmHg)	91 ± 11	89 ± 11	92 ± 11^*^	91 ± 10	88 ± 11^+^	94 ± 11	91 ± 11^+^
MAP (mmHg)	112 ± 10	111 ± 9	113 ± 10^*^	111 ± 9	110 ± 10^+^	114 ± 10	112 ± 10^+^
HR (bpm)	81 ± 14	80 ± 15	82 ± 14^*^	79 ± 15	82 ± 15^+^	80 ± 14	82 ± 14^+^
BMI (kg/m^2^)	34 ± 9	31 ± 8	35 ± 10^*^	31 ± 7	31 ± 8^+^	32 ± 8	36 ± 10^+^
Follow-up (days)	88 ± 105	87 ± 88	89 ± 112	90 ± 80	85 ± 93^+^	91 ± 106	88 ± 115^+^

*SBP indicates systolic blood pressure; DBP, diastolic blood pressure; MAP, mean arterial pressure; HR, heart rate; and BMI, body mass index. Means ± STD shown*.

* *p < 0.05 vs. White*;

+ *p < 0.05 vs. Male*.

**Table 2:** Breakdown of drug prescriptions by dosage and drug class.

		**Total**		**White**			**Black**		
	**Full population**	**White**	**Black**	**Low**	**Medium**	**High**	**Low**	**Medium**	**High**
BB	266	110	156	38	39	33	38	71	47
BBs	498	228	270	24	177	27	19	210	41
ARB	515	274	241	48	154	72	62	119	60
ACEi	1,289	560	729	110	402	48	89	561	79
CCB	2,115	470	1645	44	295	131	79	981	585
THZ	1,239	335	904	141	191	3	405	484	15
ARB + THZ	440	129	311	58	24	47	159	54	98
ACEi + THZ	1,210	303	907	128	120	55	305	367	235

*Heat mapping indicates prevalence of drug use for the full, white, or black population. Red indicates the majority in each population*.

### BP Control With Treatment

**Figure 1:**
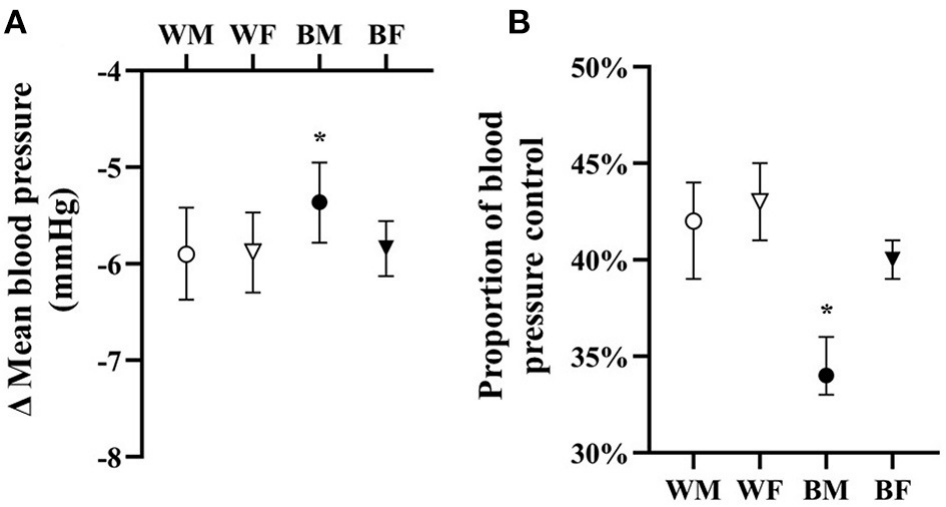
Adjusted overall responses in treated hypertensives in terms of change in mean blood pressure **(A)** and proportion of blood pressure control **(B)** with means and 95% CI shown adjusted for age, BMI, drug dose, and baseline blood pressure. *p < 0.05 vs. all.

**Figure 2:**
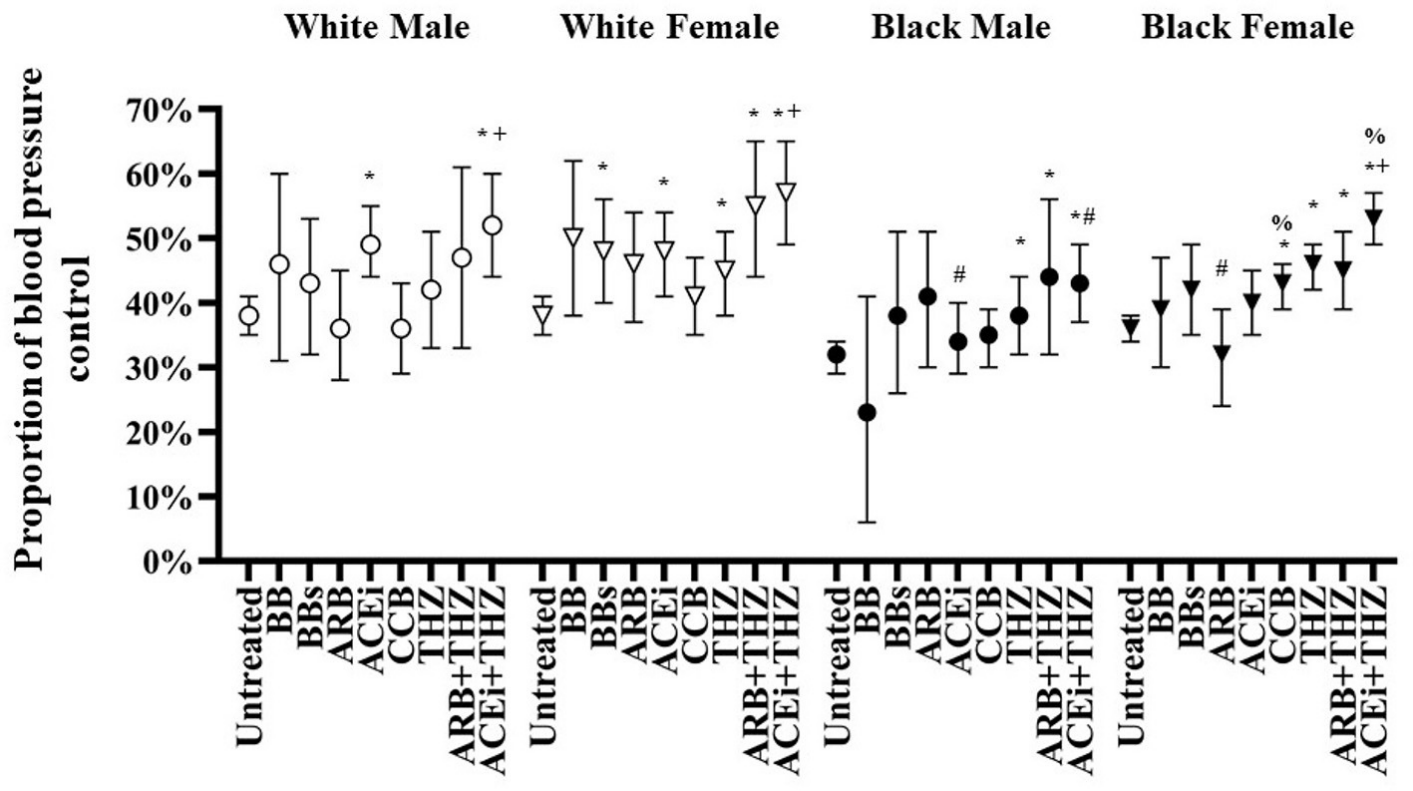
Adjusted proportion of blood pressure control after treatment with each drug class in white and black males and females adjusted for age, BMI, dosage, and baseline blood pressure (means and 95% CI shown). *p < 0.05 vs. Untreated, ^+^*p* < 0.05 vs. THZ, ^#^*p* < 0.05 vs. White, ^%^*p* < 0.05 vs. Male.

HTN treatment was associated with significant falls in mean BP and significant improvement in HTN control in all groups as compared to untreated groups overall (Figure 1). However, as compared to white males and females as well as black females, black males were associated with blunted falls in BP and lower BP control with treatment (Figure 2).

The odds of significant BP control in the whole population were seen in 6 out of the 8 drugs analyzed (Table 3). For both white and black patients, BB or ARB were not associated with improved BP control over untreated, whereas, patients taking BBs, THZ, ARB + THZ, and ACEi + THZ achieved significant BP control vs. untreated. Patients prescribed ACEi + THZ were associated with better BP control vs. THZ alone, in the whole population and within white (OR 1.70; *p* = 0.001) and black groups (OR 1.27; *p* = 0.013) (Table 3). White males only had significant BP control with ACEi and ACEi + THZ.

**Table 3:** Estimated odds ratio (95% CI) for controlling blood pressure vs. untreated at 6 months after therapy.

**Drug**	**Overall**	**White**	**Black**	**White males**	**White females**	**Black males**	**Black females**
BB	1.23 (0.93–1.62)	1.52^*^ (1.01–2.31)	1.05 (0.74–1.51)	1.37 (0.72–2.64)	1.65 (0.97–2.79)	0.65 (0.23–1.82)	1.13 (0.77–1.65)
BBs	1.36^*^ (1.09–1.70)	1.39^*^ (1.03–1.89)	1.33 (0.99–1.77)	1.22 (0.76–1.96)	1.54^*^ (1.05–2.24)	1.36 (0.77–2.38)	1.32 (0.96–1.83)
ARB	1.09 (0.88–1.35)	1.16 (0.87–1.53)	1.01 (0.75–1.37)	0.93 (0.62–1.39)	1.39 (0.96–2.01)	1.50 (0.93–2.43)	0.82^#^ (0.56–1.19)
ACEi	1.34^*^ (1.14–1.58)	1.60^*^ (1.29–1.99)	1.16^#^ (0.94–1.42)	1.62^*^ (1.21–2.16)	1.51^*^ (1.13–2.02)	1.12^#^ (0.83–1.51)	1.19 (0.93–1.52)
CCB	1.22^*^ (1.04–1.43)	1.03 (0.81–1.31)	1.28^*^ (1.07–1.52)	0.92 (0.64–1.32)	1.12 (0.83–1.50)	1.15 (0.90–1.49)	1.35^*^^%^ (1.12–1.64)
THZ	1.42^*^ (1.22–1.64)	1.26^*^ (0.98–1.62)	1.47^*^ (1.24–1.74)	1.19 (0.79–1.79)	1.33^*^ (0.97–1.81)	1.34^*^ (0.98–1.84)	1.52^*^ (1.26–1.84)
ARB + THZ	1.61^*^ (1.29–1.99)	1.77^*^ (1.22–2.58)	1.53^*^ (1.18–1.98)	1.46 (0.78–2.70)	2.02^*^ (1.27–3.21)	1.72^*^ (1.02–2.89)	1.48^*^ (1.11–1.98)
ACEi + THZ	1.97^*^^+^ (1.69–2.31)	2.06^*^^+^ (1.58–2.68)	1.94^*^^+^ (1.62–2.31)	1.82^*^^+^ (1.26–2.63)	2.27^*^^+^ (1.58–3.27)	1.66^*^^#^ (1.24–2.22)	2.10^*^^+^^%^ (1.70–2.58)

* *p < 0.05 vs. untreated*.

+ *p < 0.05 vs. THZ*.

# *p < 0.05 vs. White*.

% *p < 0.05 vs. Male*.

Adjusted proportions of BP control is shown in Figure 2. For white males, ACEi and ACEi + THZ were the only treatments with significant control over untreated. White females had significant BP control with BBs, ACEi, and THZ drugs (i.e., THZ, ARB + THZ, and ACEi + THZ). As compared with untreated, black males were only controlled with THZ drugs and black females were controlled with CCB and THZ drugs (Figure 2).

There were significant race and sex differences in HTN control. Black patients prescribed ACEi had significantly less BP control as compared to whites (Table 3). Black females had less BP control with ARB as compared to white females. Black females had significantly greater control in BP with CCB and ACEi + THZ as compared to black males, and they were associated with greater BP control with ACEi + THZ as compared to THZ alone (Figure 2).

### Changes in BP and HR

**Figure 3:**
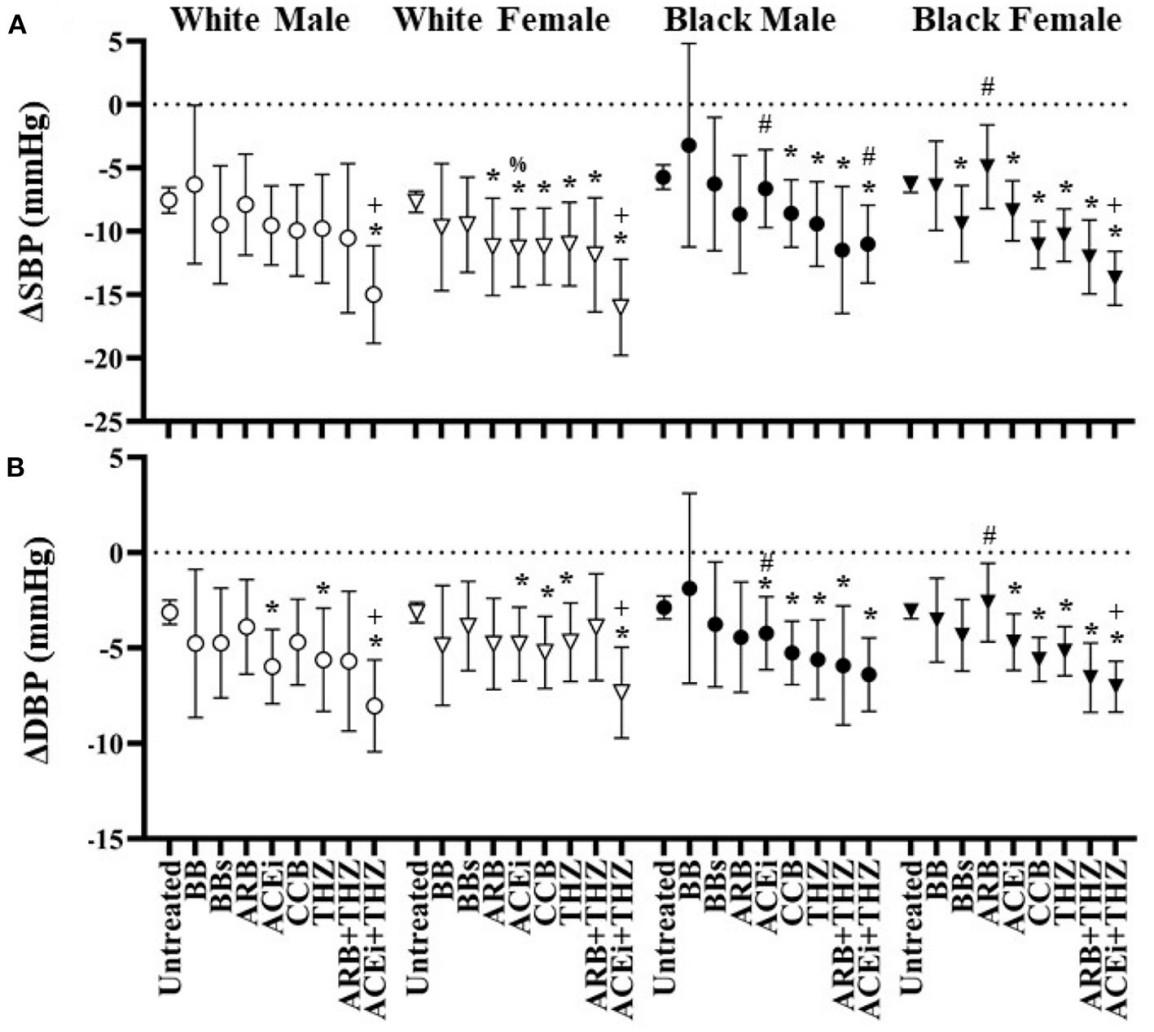
Estimated change in systolic **(A)** and diastolic **(B)** blood pressure at 6 months after each drug in white and black males and females with means and 95% CI shown adjusted for age, BMI, dosage, and baseline blood pressure. *p < 0.05 vs. Untreated, ^+^*p* < 0.05 vs. THZ, ^#^*p* < 0.05 vs. White, ^%^*p* < 0.05 vs. Male.

White males were associated with significant falls in DBP with 3 drug classes: ACEi, THZ, and ACEi + THZ (Figure 3). ACEi + THZ use in white males was also associated with significant falls in SBP. Black males, on the other hand, had lesser falls in SBP with ACEi, ACEi + THZ, and BB as compared to white males. As compared to their respective untreated group, blacks had significant falls in SBP and DBP with CCB and the drug classes that included THZ (Figure 3).

The only drug classes that did not reduce SBP or DBP significantly in white female was BB and BBs (Figure 3). Additionally, white females were the only group to be associated with a significant SBP fall in the ARB group. In black individuals, there were greater falls in BP with ARB + THZ and ACEi + THZ groups as compared to THZ alone. In whites, this association was only seen with ACEi + THZ. Finally, white females had significantly greater fall in SBP and DBP when prescribed ARB as compared to white males but less of a fall in DBP when prescribed ARB + THZ (Figure 3).

**Figure 4:**
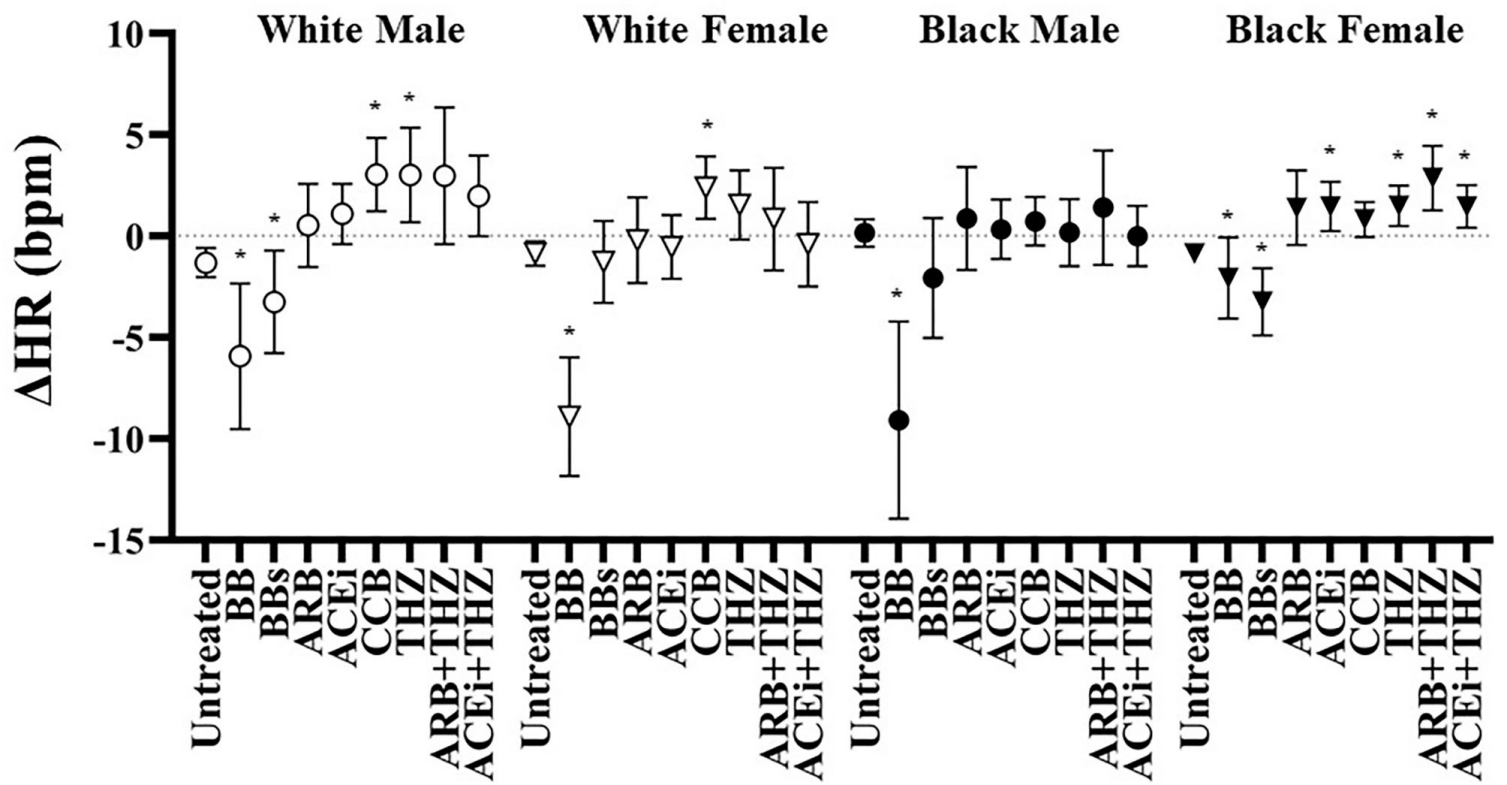
Estimated change in heart rate at 6 months after each drug in white and black males and females with means and 95% CI shown adjusted for age, BMI, dosage, and baseline heart rate. *p < 0.05 vs. Untreated.

All groups were associated with a significant fall in HR with BB use (Figure 4). On the other hand, the BBs class was only associated with reduced HR in the white male and black female groups. CCB was associated with increased HR in whites but not blacks. Increases in HR was seen with THZ in white males and all THZ drugs in black females (Figure 4).

### Predictors of BP Response

Multivariable predictors for a change in mean BP are shown in Table 4. Age, BMI, sex, and race significantly impacted the BP response to antihypertensive treatment. Additionally, although drug dose did not differ between groups, it played a significant role in predicting the BP fall (Table 4). The fall in BP was blunted in patients with increased age, higher BMI, male sex, and in black patients (Table 4). On average, the change in BP was significantly higher if a patient was prescribed a high dose.

**Table 4:** Multivariable mixed model results for the change in mean blood pressure in treated patients.

**Category**	**β-Coefficient (95% CI)**	**SE**	***P*-value**
Age	0.039 (0.026–0.053)	0.007	<0.001
BMI	0.063 (0.040–0.085)	0.012	<0.001
Male	0.65 (0.25–1.05)	0.20	0.001
Black	0.45 (0.04–0.85)	0.21	0.031
Dosage	−1.75 (−2.36 to −1.14)	0.31	<0.001

*Age and BMI coefficients indicate one-unit increases. Male coefficient is compared to females, and black is compared to white. Dosage category indicates effect from high drug dose*.

*BMI, indicates body mass index*.

## Discussion

Current treatment approaches have had minor impact on hypertension disparities. Black males have one of the lowest BP control rates (30%) and highest rates of HTN-related deaths (~70 per 100,000) (10, 11). Unfortunately, most large trials investigate antihypertensive drugs while patients are on multiple therapies that do not stay constant throughout the study (19, 20). The current study provides unique perspective from actual clinical practice and presents important findings that may be clinically useful in selecting first-line antihypertensive therapies. As we hypothesized, race played a major role in the responses to initial antihypertensive therapies. While it is well-known that renin-angiotensin system (RAS) blockers often do not provide benefit over control in blacks, our data suggests that these drugs do provide significant benefit when combined with THZ (ARB + THZ and ACEi + THZ), even more so than just THZ alone (Table 3). As discussed below, this may represent a synergistic effect due to, at least in part, to physiologically interrelated systems (i.e., the RAS system gets activated in blacks during THZ treatment). To our knowledge, this is the first evidence derived from electronic health records that demonstrate significant impacts from both race and sex in BP control. For black males and females, the >10 mmHg fall in SBP and >1.5-fold increase in HTN control after being prescribed a RAS blocker and THZ formulation is clinically relevant and could have a tangible impact in improving health disparities in Mississippi as well as the US.

Despite the pervasive use of evidence-based medicine for HTN treatment, racial disparities still remain. And according to the current data, this is independent of BMI and dosage selection. NHANES (>40,000 patient cohort representative of the whole US) reports HTN prevalence ≥40 years old to be ~60 and 44%, for black and white individuals, respectively (21). Similarly, the current data in Mississippi hypertensives demonstrate significantly higher HTN prevalence in blacks as compared to whites (57 vs. 45%, respectively) with an average age of 50 and 57 years, respectively (Table 1). Additionally, compared to white hypertensives, BP control rates in black hypertensives are significantly lower, ~42–60% (2, 13, 21). Indeed, in the current study, black male BP control rates were statistically lower overall than in white males (36 vs. 41%, respectively, *p* = 0.003). Benefits of the current study over nationwide cohorts is the applicability to socioeconomically-burdened populations who may not have the ability or desire to have frequent medical appointments for drug titration or treatment changes. Antihypertensive drug adherence rates, trust in physician care, and frequency of medical care are much lower in black patients as compared to whites (22–24). However, simplifying medical regimens and decreasing the frequency of treatment changes by prescribing efficacious first-line therapies can improve trust in health care systems and may represent an opportunity in black patients to increase drug adherence (25, 26).

Even if burdened populations are able to have sufficient physician exposure, primary care may not appropriately titrate drug regimens. Although first-line combination antihypertensive therapy has been shown to reduce mortality, many patients fail to move to combination HTN treatment despite poor responses to single drug therapy (12). For example, primary care physicians who initiated single drug antihypertensive therapy were unlikely to change or modify therapy, even when their patients had poor BP control (27–29). Indeed, 40% of hypertensive men in a large multicenter study with poor BP control were associated with unmodified antihypertensive regimens despite numerous office visits over a 2-year period (29). This highlights the crucial importance of initial antihypertensive prescription. Additionally, initiating therapy with single-pill combination drugs represents several benefits including greater efficacy at lower dose ranges due to complementary antihypertensive mechanisms, lower cost, and better compliance (30). The superior control of BP shown in black patients with drug combinations like ACEi or ARB + THZ in the current study, similar to what others have shown (31, 32), warrants further research into these combination therapies and their efficacy and socioeconomic impact in black hypertensives.

It has long been realized that BB drugs should not be used in first-line therapy in HTN treatment due to its poor efficacy and inability to reduce the risk of cardiovascular morbidity as compared to other first-line therapies (33–36). Nevertheless, both selective and non-selective BB efficacy is largely dependent on the ability to suppress renin secretion (37). For example, in a hypertensive population that included black and white patients, higher levels of baseline renin was associated with larger falls in BP after selective BB use (38). There was virtually no improvement in BP control with BB drug classes in any group, with the acception of BBs in white females (Figure 2 and Table 3). BB and BBs drugs were both associated with significant falls in HR in black females. Moreover, black males were associated with significant profound falls in HR (−9 bpm) with BB but not BBs (Figure 4). These data suggest that first-line BB prescription may not be warranted in black patients for its antihypertensive effects, but BB may be the preferred option for reducing HR over non-selective BB, especially in black males.

The decrease in BP with ARBs is more profound if a patient is white or has high activation of the RAS (1). Many clinical studies consistently report poorer BP responses to blockers of the RAS in black patients (20, 39–42). Indeed, in the current study, there were significant racial differences in the responses to blockers of the RAS (both ACEi and ARB). ACEi and ARB therapies were overall associated with poor responses in the current black population. Indeed, there was poor BP control with ACEi and ARB therapies in black males and females, respectively, as compared to the white population (Table 3 and Figure 2). On the other hand, CCB and THZ have been shown to be particularly effective in black populations (20, 41, 42). Similarly, CCB and THZ use was associated with significant HTN control and significant falls in SBP and SBP in black individuals (Table 3 and Figure 3). Interestingly, black race and female gender are significant predictors for greater BP responses to hydrochlorothiazide (43), the most common THZ drug prescribed in the current study. Coincidentally, there is some evidence that both white and black men respond favorably to another type of THZ, chlorthalidone (20, 44), which was included in the current study, but not analyzed separately.

Black patients are usually associated with lower renin levels and poor responses to RAS blockers, however, responses to RAS blockers may be synergistically improved when combined with THZ. Indeed, RAS activation that occurs after diuretic use may increase the efficacy of these combination therapies, even in patients with normal to low renin at baseline (45). In the current study, the combination therapies (ARB + THZ and ACEi + THZ) demonstrated significant BP control and efficacy, which is in agreement with the clinical recommendation of using these drugs in black patients when systolic BP is >15 mmHg above target levels (6). These data suggest that single-pill therapies that include both a RAS blocker and THZ diuretic could be considered an efficacious first-line therapy in both black and white patients in the southeast US. Studies are needed to confirm if these results are pervasive outside the Stroke Belt.

### Limitations

This study has limitations worth noting. First, the follow-up period was limited to short-term and may not be applicable to chronic responses to antihypertensive therapy such as long-term effects on heart and renal function. Many factors could have affected the overall impact of each drug's therapeutic efficacy such as medication adherence and concomitant lifestyle, behavior, or dietary changes. While these factors most likely impacted the overall efficacy of each drug, they were not monitored in the current study. There were no crossovers or change in therapy in the current study, which actually represents a potential advantage over typical clinical studies where direct effects from single drugs may be impacted from adjunctive therapies. Ideally, a significant part of primary HTN care should involve time, trial and error, and hopefully an optimized antihypertensive strategy that best fits the particular patient. While our data demonstrate racial differences in controlling BP, whether this disparity still exists with further clinical care and add-on antihypertensive therapies in this population will be the subject of future investigation. Obviously, choosing the appropriate antihypertensive therapy often takes other factors into consideration besides antihypertensive actions and BP control. For example, evidence-based benefits on heart failure and renal disease progression and function have been reported with ACEi and ARB drugs classes (46). These results suggest the use of certain monotherapies based on race and gender, but do not preclude their clinical use in any way.

## Data Availability Statement

The data analyzed in this study is subject to the following licenses/restrictions: The de-identified data is accessible to all employees of the University of Mississippi Medical Center. Requests to access these datasets should be directed to cia@umc.edu.

## Ethics Statement

Ethical review and approval was not required for the study on human participants in accordance with the local legislation and institutional requirements. For this type of retrospective study using de-identified data, formal consent was not required.

## Author Contributions

JC contributed to the conception and design of the study and drafted the manuscript. JC and SL contributed to the analysis and interpretation of data. WP created the data wrangling algorithms used to extract and organize the raw data. JC, WP, and SL revised the manuscript. All authors read and approved the final manuscript. All authors contributed to the article and approved the submitted version.

## Conflict of Interest

The authors declare that the research was conducted in the absence of any commercial or financial relationships that could be construed as a potential conflict of interest.
