# Corrigendum: Racial and Sex Differences in the Response to First-Line Antihypertensive Therapy

**DOI:** 10.3389/fcvm.2020.643289

**Published:** 2021-01-21

**Authors:** John S. Clemmer, W. Andrew Pruett, Seth T. Lirette

**Affiliations:** ^1^Department of Physiology and Biophysics, Center for Computational Medicine, University of Mississippi Medical Center, Jackson, MS, United States; ^2^Department of Data Science, John D. Bower School of Population Health, University of Mississippi Medical Center, Jackson, MS, United States

**Keywords:** hypertension, black, race, antihypertensive therapy, first line treatment

In the original article, there was a mistake in Figures 1, 2 and 3 as published. **These figures did not have any statistical markers or correct headings**. The corrected Figures 1, 2 and 3 appear below.

**Figure 1:**
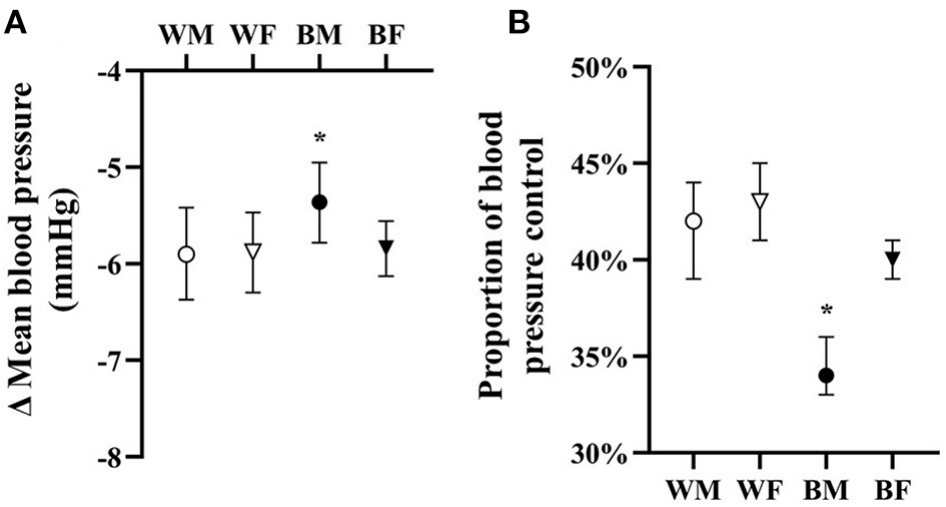
Adjusted overall responses in treated hypertensives in terms of change in mean blood pressure **(A)** and proportion of blood pressure control **(B)** with means and 95% CI shown adjusted for age, BMI, drug dose, and baseline blood pressure. *p < 0.05 vs. all.

**Figure 2:**
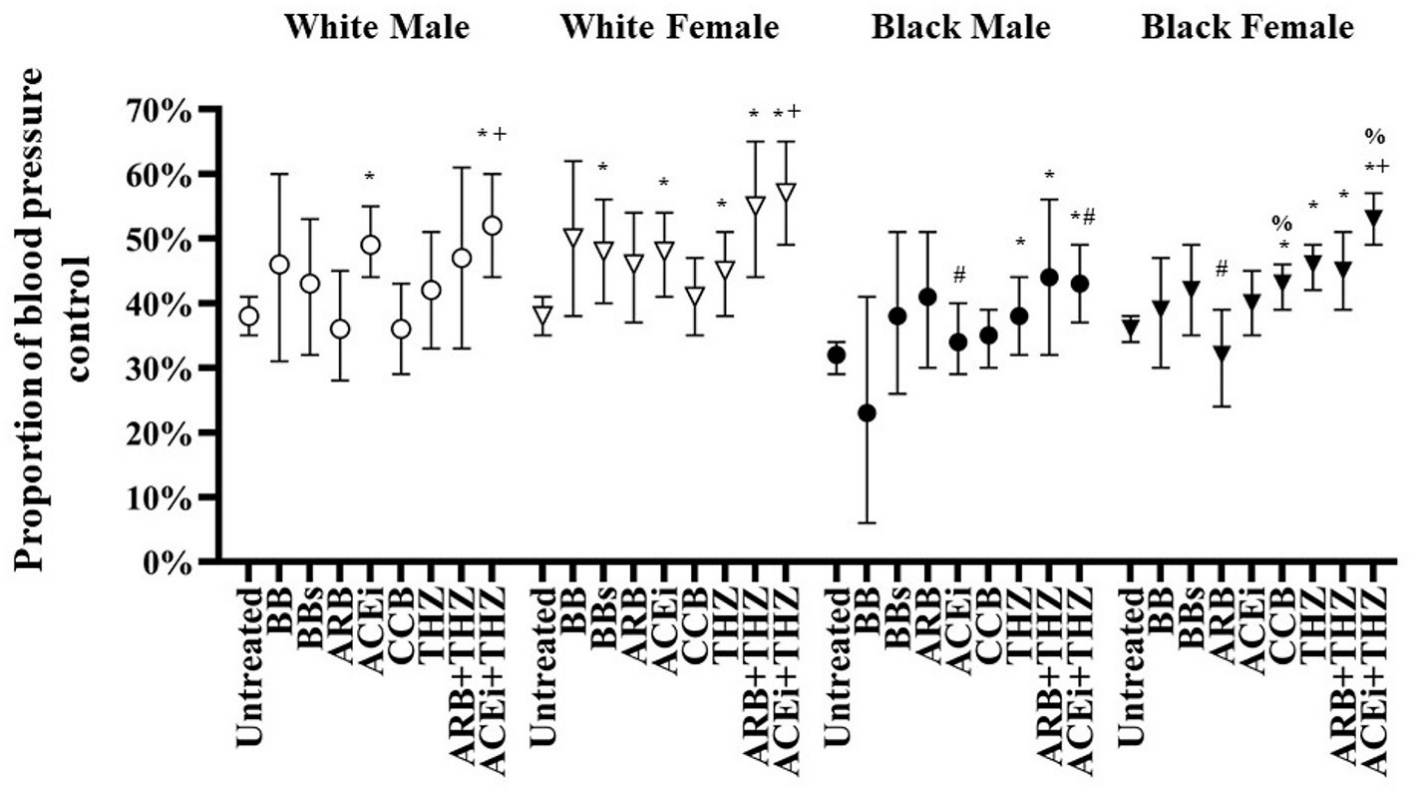
Adjusted proportion of blood pressure control after treatment with each drug class in white and black males and females adjusted for age, BMI, dosage, and baseline blood pressure (means and 95% CI shown). *p < 0.05 vs. Untreated, ^+^*p* < 0.05 vs. THZ, ^#^*p* < 0.05 vs. White, ^%^*p* < 0.05 vs. Male.

**Figure 3:**
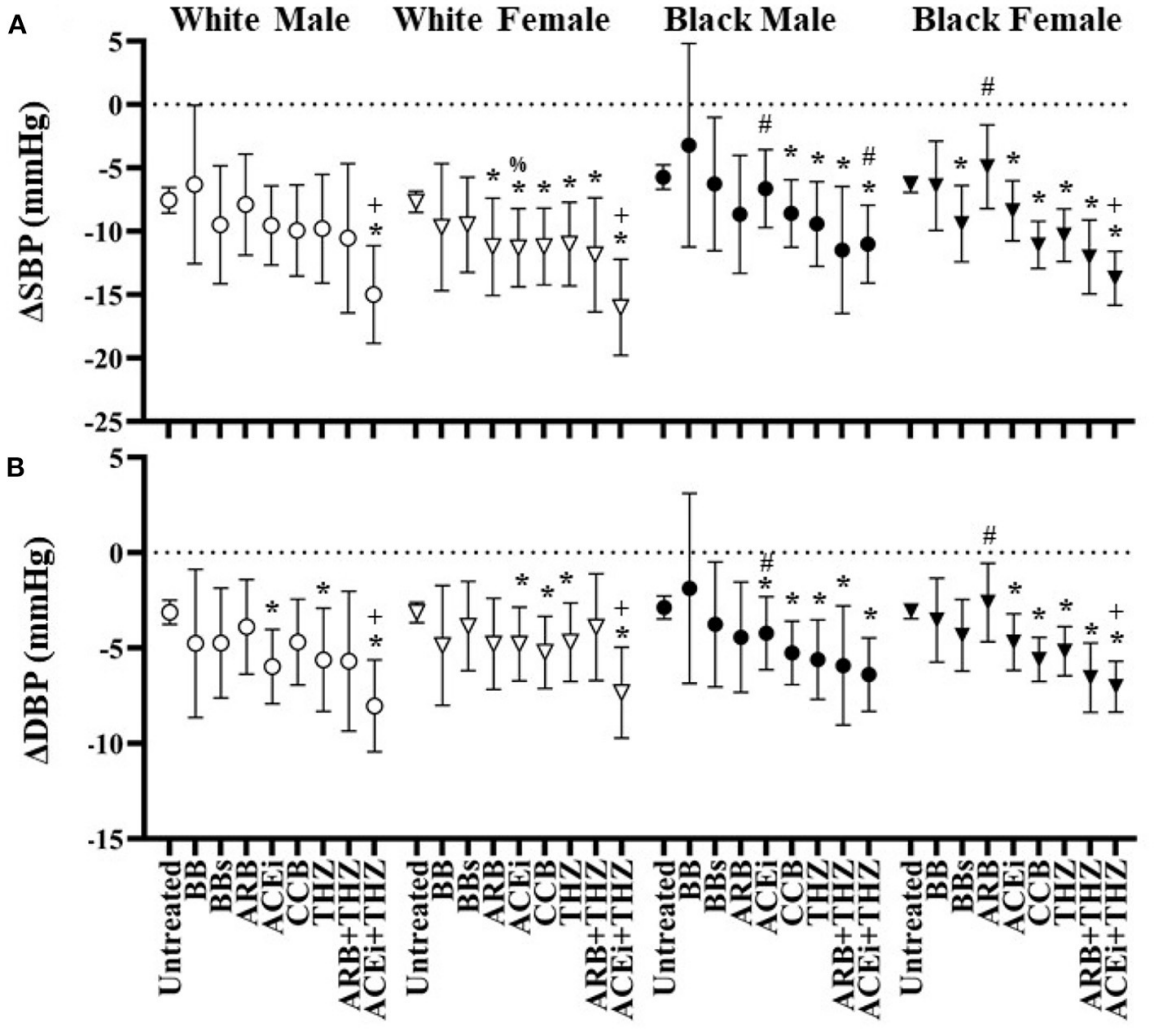
Estimated change in systolic **(A)** and diastolic **(B)** blood pressure at 6 months after each drug in white and black males and females with means and 95% CI shown adjusted for age, BMI, dosage, and baseline blood pressure. *p < 0.05 vs. Untreated, ^+^*p* < 0.05 vs. THZ, ^#^*p* < 0.05 vs. White, ^%^*p* < 0.05 vs. Male.

The authors apologize for this error and state that this does not change the scientific conclusions of the article in any way. The original article has been updated.

