# Supplementary material for: Racial and Sex Differences in the Response to First-Line Antihypertensive Therapy
Source: Front Cardiovasc Med. 2020 Dec 17;7:608037. doi: 10.3389/fcvm.2020.608037 (PMC7773696; doi:10.3389/fcvm.2020.608037)
Supplement: Supplementary file 1 [file Presentation_1.PPTX]

## Slide 1
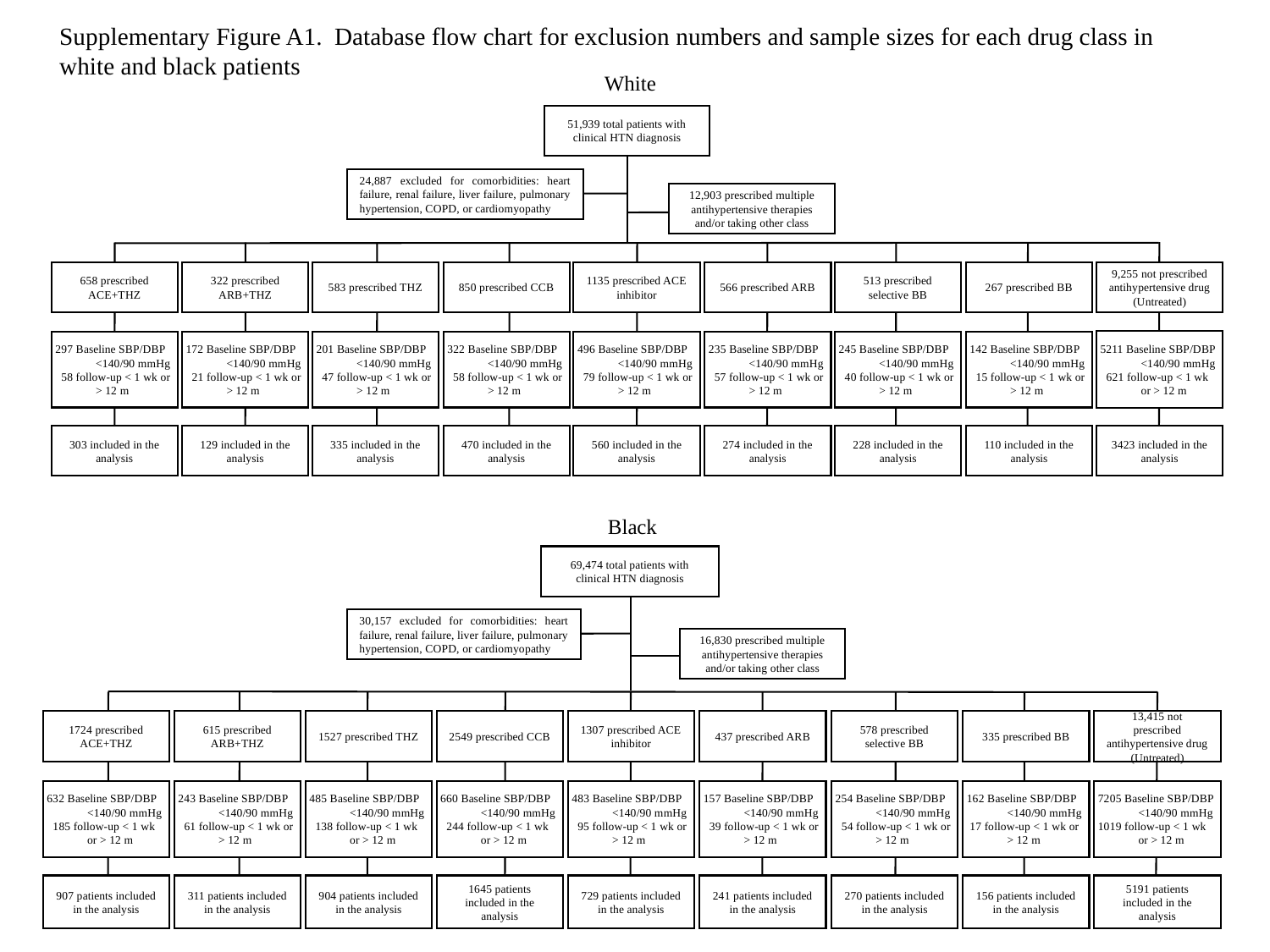

Supplementary Figure A1. Database flow chart for exclusion numbers and sample sizes for each drug class in white and black patients
White
51,939 total patients with clinical HTN diagnosis
24,887 excluded for comorbidities: heart failure, renal failure, liver failure, pulmonary hypertension, COPD, or cardiomyopathy
12,903 prescribed multiple antihypertensive therapies and/or taking other class
658 prescribed ACE+THZ
322 prescribed ARB+THZ
583 prescribed THZ
850 prescribed CCB
1135 prescribed ACE inhibitor
566 prescribed ARB
513 prescribed selective BB
267 prescribed BB
9,255 not prescribed antihypertensive drug (Untreated)
5211 Baseline SBP/DBP <140/90 mmHg
 621 follow-up < 1 wk or > 12 m
297 Baseline SBP/DBP <140/90 mmHg
 58 follow-up < 1 wk or > 12 m
172 Baseline SBP/DBP <140/90 mmHg
 21 follow-up < 1 wk or > 12 m
201 Baseline SBP/DBP <140/90 mmHg
 47 follow-up < 1 wk or > 12 m
322 Baseline SBP/DBP <140/90 mmHg
 58 follow-up < 1 wk or > 12 m
496 Baseline SBP/DBP <140/90 mmHg
 79 follow-up < 1 wk or > 12 m
235 Baseline SBP/DBP <140/90 mmHg
 57 follow-up < 1 wk or > 12 m
245 Baseline SBP/DBP <140/90 mmHg
 40 follow-up < 1 wk or > 12 m
142 Baseline SBP/DBP <140/90 mmHg
 15 follow-up < 1 wk or > 12 m
303 included in the analysis
129 included in the analysis
335 included in the analysis
470 included in the analysis
560 included in the analysis
274 included in the analysis
228 included in the analysis
110 included in the analysis
3423 included in the analysis
Black
69,474 total patients with clinical HTN diagnosis
30,157 excluded for comorbidities: heart failure, renal failure, liver failure, pulmonary hypertension, COPD, or cardiomyopathy
16,830 prescribed multiple antihypertensive therapies and/or taking other class
1724 prescribed ACE+THZ
615 prescribed ARB+THZ
1527 prescribed THZ
2549 prescribed CCB
1307 prescribed ACE inhibitor
437 prescribed ARB
578 prescribed selective BB
335 prescribed BB
13,415 not prescribed antihypertensive drug (Untreated)
7205 Baseline SBP/DBP <140/90 mmHg
1019 follow-up < 1 wk or > 12 m
632 Baseline SBP/DBP <140/90 mmHg
 185 follow-up < 1 wk or > 12 m
243 Baseline SBP/DBP <140/90 mmHg
 61 follow-up < 1 wk or > 12 m
485 Baseline SBP/DBP <140/90 mmHg
 138 follow-up < 1 wk or > 12 m
660 Baseline SBP/DBP <140/90 mmHg
 244 follow-up < 1 wk or > 12 m
483 Baseline SBP/DBP <140/90 mmHg
 95 follow-up < 1 wk or > 12 m
157 Baseline SBP/DBP <140/90 mmHg
 39 follow-up < 1 wk or > 12 m
254 Baseline SBP/DBP <140/90 mmHg
 54 follow-up < 1 wk or > 12 m
162 Baseline SBP/DBP <140/90 mmHg
 17 follow-up < 1 wk or > 12 m
907 patients included in the analysis
311 patients included in the analysis
904 patients included in the analysis
1645 patients included in the analysis
729 patients included in the analysis
241 patients included in the analysis
270 patients included in the analysis
156 patients included in the analysis
5191 patients included in the analysis

## Slide 2
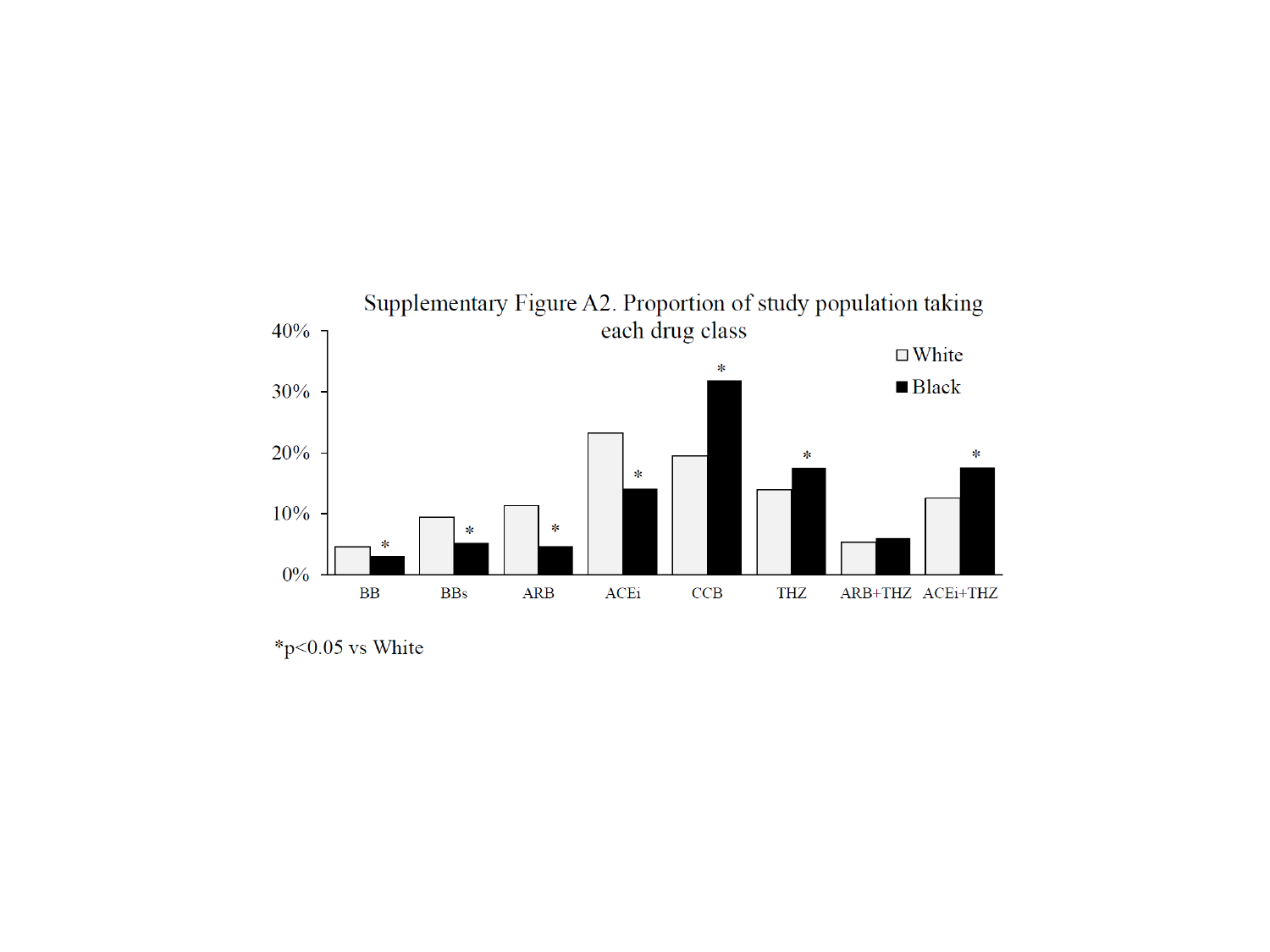

## Slide 3
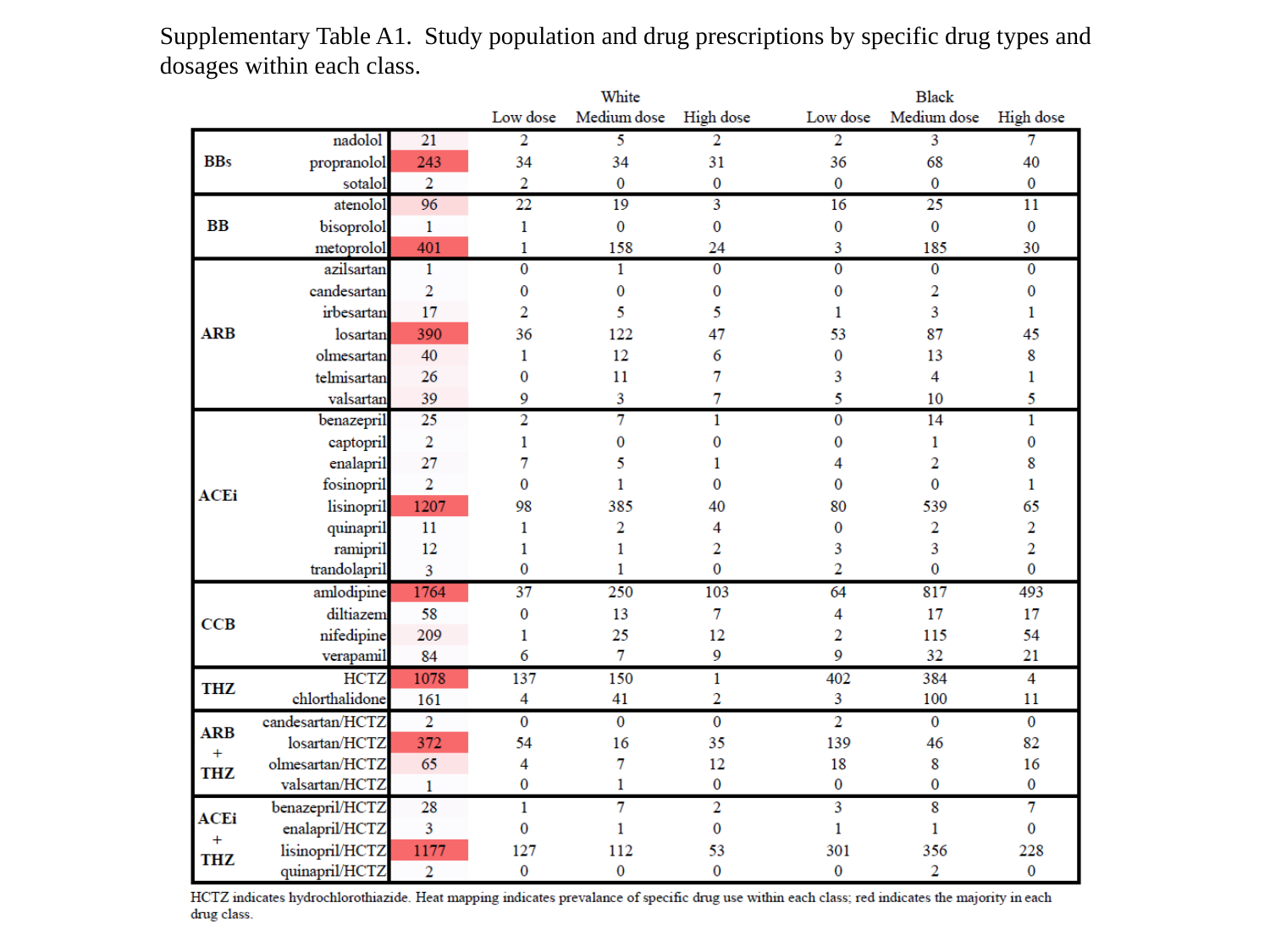

Supplementary Table A1. Study population and drug prescriptions by specific drug types and dosages within each class.
